# The application of plant-exosome-like nanovesicles as improved drug delivery systems for cancer vaccines

**DOI:** 10.1007/s12672-024-00974-6

**Published:** 2024-04-29

**Authors:** Tatiana Hillman

**Affiliations:** Biotechnology, LAL4Bsynbiotics L.L.C, Los Angeles, CA USA

## Abstract

The use of cancer immunotherapeutics is currently increasing. Cancer vaccines, as a form of immunotherapy, are gaining much attention in the medical community since specific tumor-antigens can activate immune cells to induce an anti-tumor immune response. However, the delivery of cancer vaccines presents many issues for research scientists when designing cancer treatments and requires further investigation. Nanoparticles, synthetic liposomes, bacterial vectors, viral particles, and mammalian exosomes have delivered cancer vaccines. In contrast, the use of many of these nanotechnologies produces many issues of cytotoxicity, immunogenicity, and rapid clearance by the mononuclear phagocyte system (MPS). Plant-exosome-like nanovesicles (PELNVs) can provide solutions for many of these challenges because they are innocuous and nonimmunogenic when delivering nanomedicines. Hence, this review will describe the potential use of PELNVs to deliver cancer vaccines. In this review, different approaches of cancer vaccine delivery will be detailed, the mechanism of oral vaccination for delivering cancer vaccines will be described, and the review will discuss the use of PELNVs as improved drug delivery systems for cancer vaccines via oral administration while also addressing the subsequent challenges for advancing their usage into the clinical setting.

## Introduction

**Fig. 1:**
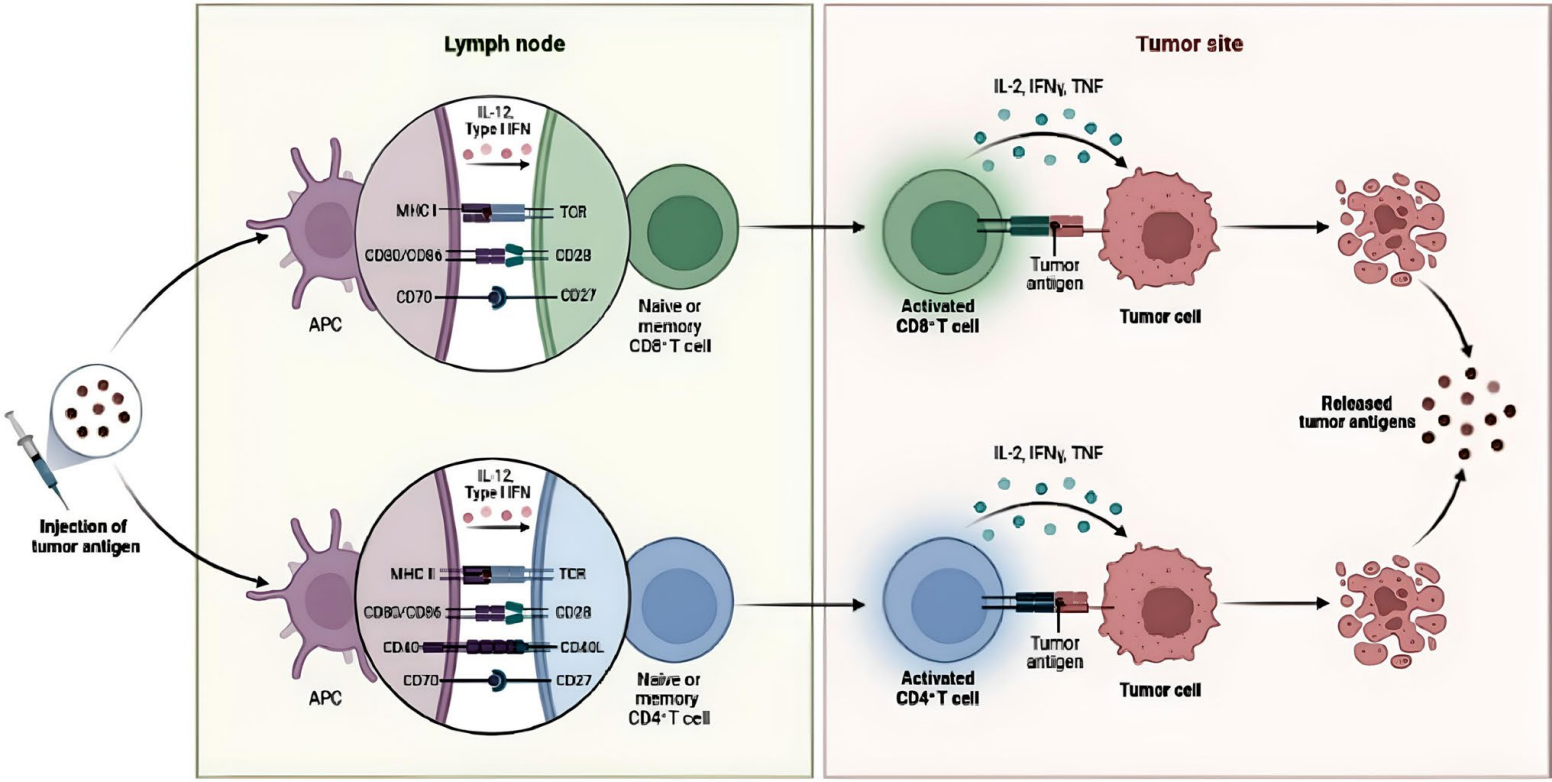
Cancer vaccines. An intravenous injection delivers the tumor antigen to the lymph nodes. The APCs present the tumor antigens to the naive or memory CD4 + T cells that activate the CD8 + T cells. Interleukins such as IL-2 and interferons such as IFN-γ and TNF are released by CD8 + T cells. The CD8 + T cells recognize and link with the tumor cells for their elimination, causing a further dispersal of released tumor antigens

Presently, cancer immunotherapy is being considered as an improved method of cancer therapy [1]. The use of vaccines is becoming a source of much promise for treating cancer [2]. Cancer vaccines function by processing and presenting antigens from tumors to antigen-presenting cells (APCs) [3] (Fig. 1). After tumor antigens are presented, antigen-specific T cells detect and eliminate cancer cells [4, 5] (Fig. 1). Vaccines are normally applied to clear infectious diseases [6]; however, vaccines can treat cancer through preventative [7] and curative [8] approaches to remedy the disease. Cancer vaccines prepare the immune cells of the host by driving the dynamics that fortify the strength of pre-existing immune responses to effectively kill local and metastatic tumors. Cancer vaccines can also create a system of memory in the immune response to inhibit future tumor development [9]. Most cancer vaccines are used to deliver antigens from tumors to antigen-presenting immune cells (APCs), more specifically from dendritic cells (DCs), for producing epitopes of the antigens to bind onto the major histocompatibility complex (MHC)—1 or to MHC-II molecular components, to be transported into the lymph nodes (LNs) for T cells to detect and become stimulated to produce an immune response. In April 2010, cancer vaccines experienced significant results and outcomes, such as Provenge Sipuleucel-T, which is an autologous cancer vaccine for the prostate [10]. Sipuleucel-T was the first human cancer vaccine approved by the FDA. Other clinical trials have successfully tested the efficacy of DNA-, RNA- and synthetic long-peptide-based [11] cancer vaccine delivery formulations. Sipuleucel-T was limited, with an increased survival rate of only 4.1 months. Other cancer vaccines have not been approved over the past ten years [12]. There are reasons for the lesser success of many clinical results of cancer vaccines, including (1) a lesser amount of efficient tumor antigens [13]; (2) a lack of enhanced adjuvant subcomponents for stimulating a significant immune response to combat tumor cells [14]; (3) long peptides have a less efficient presentation of antigens, which limits detection by CD8 + T cells [15]; and (4) much of the microenvironment of tumors inhibits the immune response, suppressing the activity of T cells to kill the tumors [16].

Nanotechnology can be used for delivering therapeutics specific for treating cancer [17], such as in the delivery of vaccines [13, 18]. Nanoparticle technology can optimize the physicochemical components to modify the ligands targeted. Nanoparticles can gather internally in LNs, and APCs can efficiently internalize the NPs. Second, nanoparticles can prevent the degradation of their cargo by lysosomes, which can improve the presentation of antigens by the MHC-I process [19]. Many nanoparticles can more directly deliver cargoes into the cell cytoplasm via endocytosis, which fine-tunes the presentation of antigens. Peptides [20], DNA [21], and small molecules [22] could orient the binding of lipid-based nanoparticles with the cell's plasma membranes to deliver the cargo directly into the cytosol. Biomimetic fusion liposomes, which are generated by binding the liposome with an inactivated Sendai virus, deliver molecules more directly into the cytoplasm and produce a cytotoxic T lymphocyte (CTL)-stimulated response [23]. Third, nanoparticles can mobilize many different types of antigens and adjuvants to enhance the immune response by a combined delivery of the necessary components to the simultaneous and shared APCs. Bacterial derivatives delivered vaccine vectors [24]. Virus-like particles (VLPs) and virosomes have delivered cancer vaccines due to their innate tropism and their great effectiveness at transiting across biological barriers, and VLPs also produce a significant immune response [25].

However, most vaccinations are administered intravenously, via subcutaneous or intramuscular injections, in which these injections for vaccination cause a lower rate of vaccinations over time [26]. Other routes for administering vaccines are needed for further examination to maximize the compliance from patients to receive immunizations and booster shot injections [27, 28]. An oral route of administration is a favorable route for vaccinations, but because of the degrading environment of the gut, an oral route is challenging for delivering therapeutics, such as insulin, chemokines, and vaccines [29]. The oral route of administration is still the most auspicious because of its potential for self-administration; it is safer, increases compliance from patients, and has a facile distribution when compared to intravenous injection therapies [28, 30]. Consequently, different strategies for the oral delivery of therapeutics have been investigated and examined, but the oral route is unsuitable for the direct delivery of proteins and peptides, in which new developments in drug delivery systems are altering the approaches to vaccine delivery, including the oral delivery of mRNA vaccines [31]. In oral delivery, the gut lymphoid tissues, present throughout the intestines, have membranous (M) cells on their surface, which offers a direct passageway of entry for antigens into the immune system wherein the gut provides a sufficient access point to immune cells. Additionally, many research scientists face challenges when investigating new therapeutic strategies, such as issues with delivery, targeting the specificity of the tissues, contesting off-target effects, increased toxicity, expensive production costs, and safety issues. Plant-exosome-like nanovesicles (PELNVs) can address many of these challenges because they are innocuous and environmentally friendly for delivering nanomedicines [32]. Compared with synthetic nanoparticles, which are highly immunogenic, cytotoxic, and difficult to manufacture, PELNVs can be less immunogenic, are not detected by the immune system and have longer periods of systemic circulation with an increased level of bioavailability [32]. PELNVs are slightly different from mammalian ELNVs because PELNVs do not store zoonotic or human pathogens. PELNVs are more favorable than mammalian cell-derived ELNVs because they are not immunogenic and are innately innocuous. PELNVs are sufficiently processed with maximized uptake at the target site, can effectively deliver therapeutics and are frugal to produce [33].

PENLVs are more efficacious than synthetic nanoparticles because they are more innocuous, have less immunogenicity, have improved uptake by cells, and have an increased level of stability when traversing the GI tract (GIT) with amplified targeting potential [34]. However, there is a lack of reviews of PELNVs because there are a few articles of original research in this field of PELNV study without any of the available studies detailing all the properties of PELNVs [32]. Nevertheless, PELNVs have been demonstrated to be significantly resilient to degradation by gastric enzymes with changes in pH present in the gastrointestinal tract, in which PELNVs could facilitate improved delivery of cancer vaccines via oral administration. The objective of this review is to demonstrate the potential of PELNVS to deliver cancer vaccines via an oral route of administration. This present review will provide a brief background for the different drug delivery systems of cancer therapeutics, including cancer vaccines, describe the benefits of oral vaccine delivery for treating cancer, present the immense potential for utilizing PELNVs as an improved drug delivery system for cancer vaccines via oral administration, and discuss the potential challenges of using PELNVs for drug delivery.

### Cancer vaccines delivered by synthetic nanoparticle carriers

By targeting the peripheral immune system for a response, a strong immune response can occur in LNs. LNs are pertinent sites for presenting antigens to T cells and for the targeting of tumor cells. The use of vaccines to target the LN for eliminating tumors could be more effective for stimulating T-cell responses by distributing a sufficient amount of antigens. However, for "cold" tumors that do not produce many immune responses, vaccines may not be as effective for creating an anticancer immune response. An antitumor response can be significantly induced by endogenous and exogenous cancer vaccines [10]. Many different immune cells are stored in the LNs, such as CD4 + helper T cells, B cells, APCs, and CD8 + T cells. Delivering antigens to APCs is the main occupation for a cancer vaccine since the presence of the antigens through APCs initiates the adaptive immune response [35]. The three main approaches for targeting LNs with nanovaccines include (1) modifying the physicochemical properties to allow passive transport of the nanoparticles into the LNs; (2) altering the nanovaccine with APC-based ligands or antibodies to generate active transport [10]; or (3) being transported while bound to an endogenous molecule or cell. Vaccine delivery systems with a size of 10 to 100 nm can easily cross the intestinal epithelium into the LNs by passive transport via lymphatic capillaries [10]. Nanoparticles of a larger size, such as 500 to 2000 nm, become stagnated in the stroma, in which their transport depends on cellular uptake [10]. Adding PEGs can produce more stable cancer vaccines for a more direct LN-targeting approach, in which the PEGylated nanoparticles stimulate an anti-PEG response of immunoglobulin M antibodies, leading to their rapid clearance from the blood [10]. Erythrocyte membrane-camouflaged nanoparticles can mimic red blood cells (RBCs) and accomplish a longer period of blood circulation compared to the PEG-coated nanoparticles [10]. Surface charge can also influence the uptake of nanoparticles by APCs and affect the targeting of LNs. Nanoparticles that have a negative charge cannot become immobilized in the stroma and the interstitium for transport into the LNs. Positively charged nanocarriers become more phagocytosed by DCs after fully transitioning into the LNs [36].

Hence, to produce a potent immune response, a a balanced design between LN targeting and uptake by APCs is needed. Furthermore, adding ligands nanoparticles can allow more active targeting of LNs via ligands specifically binding to APCs. Receptors termed the C-type lectin receptors are ligands that can specifically target APCs, which have structural homologies similar to carbohydrate sugar molecules [37]. With the use of cell labeling and bioorthogonal chemistry, it has become possible to deliver exogenous immune-regulating materials into LNs [10]. Engineering unnatural sugars [38] and orienting lipids into cell membranes [39] cause persistent labeling of DCs and lymphatic endothelial cells, respectively. When bound to different pathogen-associated molecular patterns (PAMPs), a few pathogen formations, including outer membrane vesicles (OMVs), target the LN or other immune cells [40]. An 'albumin hitchhike' includes the process of binding vaccine components to endogenous albumin for targeting the LN. Antigens that become altered with lipophilic albumin-binding domains have the propensity to collect and become stored in the LNs after injection and an in situ linkage with endogenous albumin. The "albumin hitchhike" when compared to traditional vaccines initiated a 30-fold increase in the priming of T cells, leading to a maintained suppression of TC1 tumors [41]. This approach was applied to CAR-T therapy when binding the ligand from a CAR to a polymer-lipid tail. The CAR ligands bound readily link with albumin to target LNs and become inserted into the APC membranes to enhance the CAR-T therapy. This approach led to a significant decrease in the tumor size, and the approach could be applied to assist with the proliferation of CAR-T cells toward many various tumor targets [42].

### Exosomes as a drug delivery system for cancer chemotherapeutic agents

Mammalian-based exosomes can also enhance the delivery of cancer therapeutics. Exosomes are membrane-bound vesicles that are discharged from cells into the extracellular matrix [43, 44]. Nanoparticles are synthesized internally in the cells inside MVBs, leading to the production of intraluminal vesicles in MVBs [43, 44]. These pre-exosomes are released from the cells after the MVBs fuse with the cellular membrane [43, 44]. MVBs secrete ILVs, but they fuse to lysosomes with ILVs, and their cargo becomes disposed of for recycling [43, 44]. MVBs can bind with autophagosomes in the process of autophagy to create amphisomes, which are hybrid vesicles that can fuse with the cellular plasma membrane to excrete exosomes with their cargoes [45, 46]. There are many different molecules expressed on the membrane of MVBs that assist with the loading of cargo, the budding of the membrane, and the secretion of ILVs into MVBs [43, 44]. For example, the ESCRT-linked mechanism has four molecular complexes that assist with biogenesis and the loading of ILVs with MVBs [43, 44], utilizing ATP [60, 61]. Rab proteins bound to MVBs mediate the mobilization of MVBs [43, 44]. The Rab proteins Rab7, Rab8, Rab11, Rab27, and Rab35 assist in modulating the exosome pathway [45–48]. After the release of the exosomes, the exosomes transit to the target cells. EVs can influence recipient cells by endocytosis, direct fusion, and through a receptor‒ligand dynamic interaction, which triggers alterations in the biological processes of the cells [49]. More research is necessary to find more proximate processes of how exosomes are produced.

Exosomes accumulate their cargoes from the cellular membrane, cytoplasm, Golgi apparatus, and endosomal subsections of the cell [43, 44]. Exosomes have a biconcave and spherical structure when viewed under an electron microscope [50]. The vesicles have a thickness and viscosity of 1.08–1.19 g/mL. EVs also have biomarkers, such as CD9, CD63, CD82, CD81, Alix, and Tsg101 [51, 52]. Exosomes carry thousands of biomolecules, such as RNAs, proteins, and lipids. A better understanding of the innate characteristics, purity, and biogenesis of exosomes is pertinent for many downstream experiments. For example, exosomes extracted from stem cells may help integrate the procedures of regeneration with physiology, as the EVs isolated from cancer cells can assist with protocols specific for pathogenesis [53, 54]. Exosomes can deliver therapeutics and have been investigated in preclinical and clinical settings [55]. Exosomes are favorable candidates for translating many drug-delivery system formulations into the clinical setting [55]. Exosomes are similar to liposomes due to their complex phospholipid structure. Exosomes can be isolated from bodily fluids and cells. Exosomes have complex biomolecules on their surfaces that may mediate the targeting of cells and tissues [56, 57]. The proteins on the surface of the phospholipid membranes of the exosomes have encryption that guides their interaction with their target sites. There is a debate that still exists about whether exosomes are more effective than liposomes [58]. Therapeutic drug molecules, nucleic acids, and proteins can be loaded into exosomes [55, 59] by direct exogenous methods, in which the drugs are inserted into the extracted exosomes by many different approaches. Indirect methods can be used to load exosomes as well that include endogenous methods where the progenitor cells are loaded with the therapeutic agents to passively transfer the drug into the EV during biogenesis. The cells can also become modified genetically to express the cargoes of RNAs, receptors, and proteins to transit to the EVs.

### Plant exosome-like nanovesicles (PELNVs) as potential drug delivery systems for chemotherapeutic agents

However, the coatings of nanoparticle carriers with PEGs usually lengthen the circulation time and assist with immune tolerance, but many of these coatings can inhibit the interaction between the nanoparticles and the targeted cells, which reduces the biodistribution of the therapeutics [60]. Continued exposure to PEG-coated liposomes can form anti-PEG antibodies, rendering the therapy less efficacious [61, 62]. Synthetic nanoparticles can only deliver therapeutics without any natural therapeutic capabilities. However, PELNVs have many innate therapeutic abilities, such as antitumor effects, they are regenerative, and have anti-inflammatory properties. PELNVs can deeply penetrate tissues, have a negative charge that contributes to their lengthened circulation [63], have a similar morphology to the plasma membrane, and have a more stable physicochemistry under different pH conditions and temperatures [64, 65]. When compared to liposomes, they can target tumor tissues at a tenfold higher level, which shows that PELNVs have a better capability for tumor targeting [66]. PELNVs differ from mammalian and bacterial-derived ELNVs because PELNVs decrease the potential toxicity of undesired genetic or protein transfers, which can diminish the immune response [67]. PELNV-derived nanoparticles can successfully transit the blood‒brain barrier (BBB), which is different from and unlike synthetically engineered liposomes [68]. The lipid bilayer of PELNVs covers and securely encapsulates the cargo, preventing the enzymatic degradation of the cargo by proteases and nucleases [69].

PELNVs are a promising drug delivery system [31]. PELNVs can be utilized for colon-targeted delivery through oral administration [70] because PELNVs have an innate capability for improving the bioavailability of bioactive molecules by maintaining the stability and security of the loaded cargo [71, 72]. A study showed that ELNVs were able to block lysis by detergents more than other extracellular vesicles (EVs) [73]. PELNVs can avoid degradation by digestive enzymes, such as pepsin, pancreatic enzymes, and bile salts. The resistance of PELNV to these enzymes assists in preventing the decomposition of their loaded cargo by the volatile environment of the GI tract, allowing secure delivery into the colon. However, a significant understanding is still lacking of how PELNVs can transit such a harshly conditioned GI tract environment while safely delivering and securing their loaded cargo into colonocytes. For example, grapefruit-derived ELNVs were loaded with methotrexate to target macrophages in the intestines. After oral administration of the methotrexate-loaded grapefruit-derived ELNVs, the conjugated therapeutic efficiently targeted the F4/80 + macrophages present in the intestinal lamina propria [74]. After macrophage uptake of these conjugates, colitis and the expression of the proinflammatory cytokines TNF-a, IL-1b, and IL-6 were reduced in mice [31]. Methotrexate side effects were significantly decreased when delivered by grapefruit-derived ELNVs versus free methotrexate.

PELNVs can naturally decrease inflammation while providing targeted delivery into macrophages for treating autoimmune illnesses and colon cancer [75]. PELNVs can also target intestinal cells where they target macrophages [76]. PELNVs are natural transporters lacking potential side effects at systemic and organ levels, including PELNVs extracted from organic vegetables and fruits that are normally consumed by most individuals throughout the world. PELNVs should be extracted from organic vegetable and fruit sources to prevent any contamination from pesticides with the exosomes [77]. PELNVs have limited forms of toxicity and reduced responses of immunogenicity when compared to exosomes extracted from mammalian cells. PELNVs isolated from nontoxic plants can be extracted from large amounts of green sources [78]. PELNVs extracted from grapefruits have delivered drugs into different cells without displaying cytotoxicity and did not induce inflammation through cytokine stimulation [79]. PELNVs derived from ginger that inhibited the growth of tumors in mice were stable when in different pH values, biodegradable, in which these ginger-PELNVs can be used in a new oral drug delivery system, and were less toxic than synthetic lipid nanoparticles. There is profound interest in using PELNVs as drug-delivery systems because they do not enter filtering via organ sequestration and do not cause systemic toxicity [80].

### Extracting and cargo loading of PELNVs

**Fig. 2:**
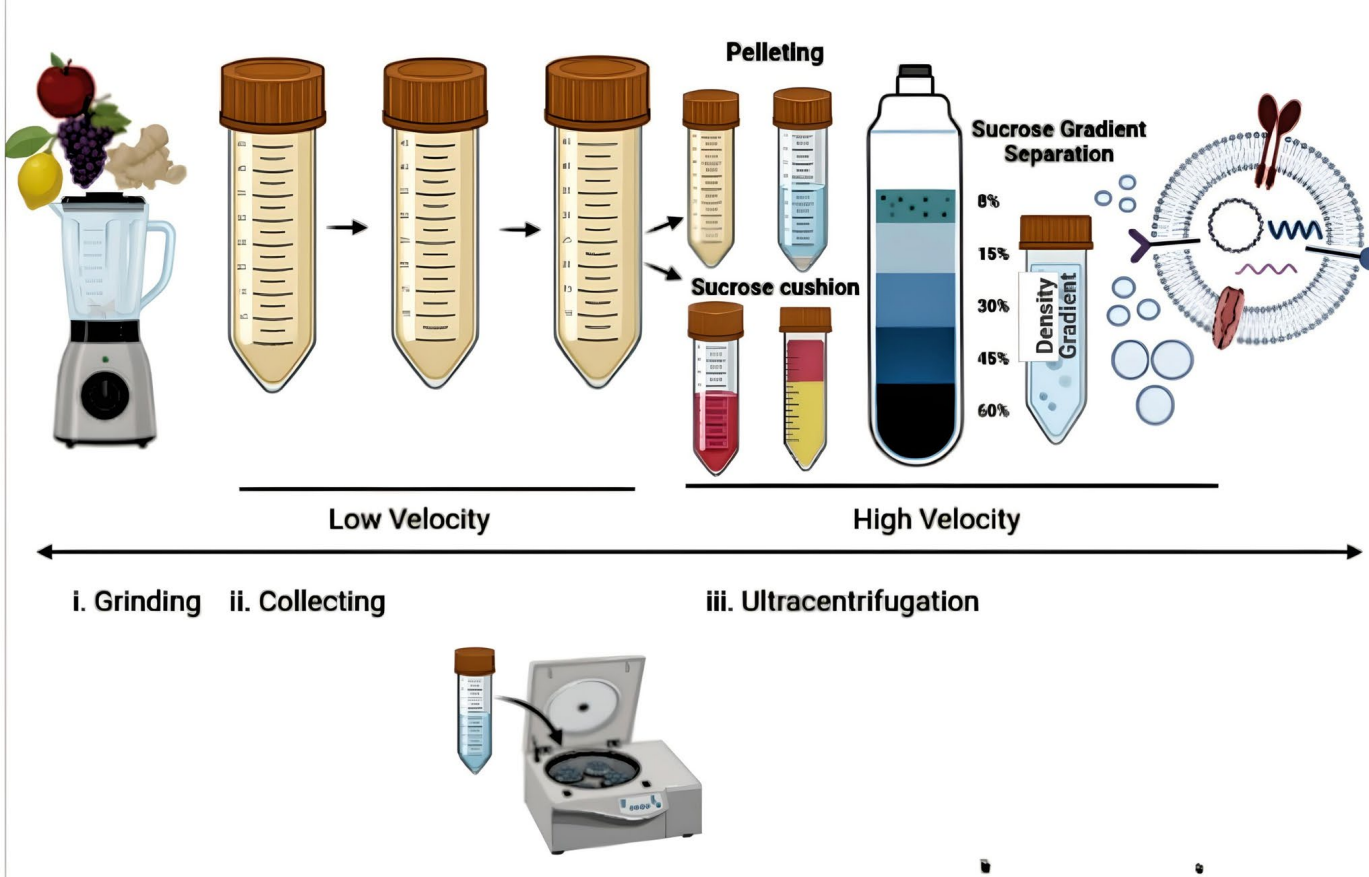
Isolation and reassembly of PELNVs. The figure presents the process of isolating PELNVs from fruits, such as apples, grapes, lemons, and ginger, through low-to high-velocity ultracentrifugation and the use of a sucrose gradient

Since 1967, there has been ample evidence that proves PELNVs exist. In earlier studies, the presence of multivesicular bodies (MVBs) in plant cells was examined [81, 82]. Currently, the shape and morphology of the PELNVs were observed to be related to cell differentiation, leading to the cell wall's high viscosity [83–85]. The development of PELNVs is actuated when the trans-Golgi network or an endosome form progresses into mature multivesicular endosomes (MVEs) or MVBs. The intraluminal vesicles (ILVs) consist of RNA, such as mRNAs, microRNAs (miRNAs), and many other types of noncoding RNAs with DNA and lipids internal to the MVBs. MVBs link with the plasma membrane to guide the release of PELNVs [86]. Differential ultracentrifugation is used to isolate and purify ELNVs [87–90]. This strategy functions based on the size and density of the particles [91]. Plant juices are the source of PELNVs that are centrifuged at gradually elevated speeds. More lengthier centrifugations take place after each subsequent centrifugation to separate the high-density particles with the pellets being disposed of as waste (Fig. 2). The supernatant was then centrifuged at a higher speed of 100,000 g to collect the PELNVs as a pellet. The pellet was resuspended and washed in phosphate-buffered saline (PBS) [92]. The acquired PELNV has impurities of nucleic acids and proteins [93]. To continue the purification process, the suspension was homogenized and placed in a sucrose gradient of ultracentrifugation (SGC), including an 8%, 15%, 30%, 45%, and 60% gradient, at a speed of 150,000 × g for 120 min [32].

The sucrose gradient is the most important step for retrieving the purest forms of PELNVs. The PELNVs remain suspended in sucrose relying on the g-force of sedimentation and the different speeds of the sucrose gradients [94]. PELNVs obtained from the 30 to 45% layers are the most favorable and distinguished particles. The discarding of the pellets after the 100,000 g UC with an additional UC at 135,000 g eliminates the presence of other EVs. However, after the use of many UCs to achieve the highest purity of PELNVs, the yield of PELNVs is lowered. Furthermore, the use of high levels and speeds of centrifugation may damage the structural stability of the PELNVs, causing agglomeration [32]. The use of a high-density iso-osmotic cushion at the bottom of the centrifuge tube may improve the yield and quality of the PELNVs. There are also PELNV isolation kits that are available that are used by researchers. However, using the kits has been shown to engender co-precipitation with other EVs and PELNVs, including RNA‒protein complexes [95]. Re-engineering the PELNVs is a more arduous effort than preparing more uniform-sized PELNVs since they have many different sizes ranging from 50 to 500 nm, which is a major challenge for their wider use in drug delivery [32]. The loading of the drug is also another concern for the use of pure PELNVs. It is difficult to prepare uniform-sized nanoparticles while ensuring secure loading of the therapeutic [96]. Therefore, researchers apply the Bligh and Dyer method, which is a liquid-to-liquid extraction technique, to extract nanolipids from PELNVs, and then, a 200-nm liposome extruder processes the extracted nanolipid, re-engineering them into more uniform-sized nanocarriers termed PELNV-derived nanovectors (PEVS) [97].

Although PELNVs innately carry lipids and other cell-linking molecules, such as phosphatidic acid (PA), which helps the PELNVs to bind to the targeted cells [98], PELNVs can achieve most of their targeting by having a more natural propensity of biodistribution. The surfaces of PEVs can also be modified, which can increase the level and intensity of their targeting capabilities [99]. Bioactive compounds of molecules can modify the surfaces of PELNV-based nanocarriers to guide an improved link with cellular surface receptors and targets. The surfaces of PELNV-based nanocarriers can be modified to more specifically bind to tumors, increasing the antitumor effects of these nanocarriers [100]. For instance, ginger-derived ELNVs modified with a tail of pRNA-3 WJ-folic acid (FA) displayed the ligand and delivered genes at the site of tumors [101]. The incubation technique can modify the surfaces of PELNV-based nanocarriers, which implement the processes of hydrophobicity, diffusion, and electrostatic interactions [102]. Moreover, a few ligands oriented onto the surface of PELNVs can create tags for bioimaging methods [102]. Through the use of click chemistry, macromolecules can conjugate with small molecules, such as imaging components linked to the surfaces of PELNVs through covalent bonding [103]. Through modification of the PELNV surfaces, the amplification of delivered nucleic acids occurs. Adding polyethyleneimine to grapefruit-derived nanovectors heightened the gene delivery to brain tumors and decreased the level of cytotoxicity of the polyethyleneimine [33]. By modifying the lipid bilayers of PELNVs with cell-penetrating peptides, the cellular uptake increased with amplified penetration of tissues [104].

Therapeutic drugs conjugated to the surfaces of PELNV-based nanocarriers augment their therapeutic effects. PELNVs can encapsulate cargo such as drug molecules, proteins, expression vectors, siRNA, and DNA. PELNVs can be prepared from plant sources and then loaded with drug molecules directly. To load cargo into PELNVs, passive and active loading mechanisms are feasible for use [105]. For passive cargo loading, an applied incubation method incubates the drug molecules with the PELNVs at a specific temperature. The diffusion action and lipophilic interaction occur between the drug molecules and the lipid bilayers of the PELNVs while implementing the passive encapsulation method [106, 107]. In active loadings, the use of sonication slightly disfigures the PELNV membrane structure for the cargo to diffuse into the PELNVs. After the sonication process, the PENLVs return to their original structural form after loading the cargo. Active loading is superior to passive cargo loading by 11-fold [108]. PELNVs have a negatively charged surface that interacts mostly with positively charged drug molecules, such as doxorubicin, and complements drug loading during the sonication process [109]. Negatively charged molecules and FA may have a more difficult cargo loading process. Data reports show that lipophilic cargos can overcome the surface-based electrostatic forces of PELNV- based nanocarriers. Other studies have provided evidence for siRNA and DNA, which have a negative charge, but nucleic acids can still produce PELNVs with the expected therapeutic effects [110]. Although PELNVs have many advantages, the cargo loading process damages them, which causes some distortion and requires a novel solution for this issue.

Many of the biomolecules that PELNVs consist of may need to become unloaded to provide more space for the loading of other therapeutic molecules. However, PELNVs have not been examined for potential unloading procedures [32]. RNAs still have many difficulties when loaded into PELNVs. Many studies have shown that electroporation can assist with cargo loading of RNA into PELNVs with a highly efficient loading capacity with an increase in voltage and an improvement in the PELNV-to-siRNA ratio during the process of electroporation [111, 112]. However, electroporation cannot load mRNA and DNA into the PELNVs at a similar efficiency as the smaller RNAs [113]. Passive loading with the transfection of target cells with cargo through viral or nonviral nanovectors can increase the cargo loading of RNA into PELNVs. All of these methods may offer solutions for the many present challenges related to RNA loading into PELNVs.

### PELNVs and cancer

Grapefruit-based nanovectors effectively delivered FA and paclitaxel into CT26 colon cancer cells and human SW620 colon cancer cells in mice with severe combined immunodeficiency (SCID). Researchers intravenously administered these nanovectors to mice. The nanovectors did not cross from the womb of pregnant mice into the placental regions when they received intravenous injections; as a result, grapefruit-derived nanovectors can treat pregnant women by delivering therapeutic drugs [34]. An inflammatory chemokine receptor-enriched plasma membrane used as a coating modified the grapefruit-based nanovectors, and then, these coated nanovectors effectively targeted the CT26 tumor and the 4T1 tumor in the breast of the mouse models, delivering doxorubicin with curcumin to the site of the tumors [31]. PELNVs can apply their innate antitumor capabilities with their significant potential as a drug delivery system since the leukocyte function-associated antigen-1 (LFA-1) and CXC chemokine receptor 2 (CXCR2) found on the plant-derived nano vector surfaces attenuated the inflammation of the tumor target site [100]. However, the therapeutic effect of the PELNVs released depends on the source of the plant. For example, naringin is a central flavanone found in grapefruit [114] that can become processed via hydrolysis by the microbiota present in the intestines. Naringin becomes hydrolyzed into naringenin, which has tremendous antitumor effects [114, 115].

PELNVs can suppress the expression of the cascade of proteins that lead to the development of tumors. For instance, PELNVs can act as antitumor agents by impeding the expression of cyclin D1 mRNA and then triggering the activation of cyclic guanosine monophosphate (cGMP)-dependent protein kinase, which further inhibits the development of the tumor by suppressing T-cell transcription and signaling [116]. PELNVs can also be highly apoptotic and suppress angiogenesis by inhibiting cytokines in cancerous cells while neglecting healthy cells [117]. For these reasons, PELNVs can be combined with chemotherapy to treat and eliminate cancer in the near future.

### An oral administration of a cancer vaccine

Oral delivery is the most favorable type of administration, but the digestive system presents many innate challenges, causing only a small number of approved vaccines that are fit for oral delivery. Every section of the GI tract poses a different obstacle to engineering an effective vaccine [118]. With most pathogens, approximately 90% enter through the mucosal pathway, which includes the GI tract, respiratory tract, and urogenital pathways. The mucosal systems are more vulnerable to infections than the skin because of the exposed mucosal membrane layers [26]. Therefore, an immune response in the mucosal pathways would be more favorable as a first barrier of defense to fully prevent and clear infections. A pertinent immunoglobulin called secretory IgA (SIgA) is necessary for blocking the passage of pathogens past mucosal sites [119].

Nanogene delivery systems formulated for oral delivery were designed to accomplish many different actions. First, they enhance the transport of genes across the intestinal epithelium by improving diffusion through enterocytes and M cells. Second, they have a high accumulation in the LNs by intestinal transport through the lymphatic system and are efficiently facilitated by macrophages in the organs and tissues of the lymphatic system [120]. Oral delivery of genes is a safer and easier route of administration, specifically for patients with longer-term medication treatments. Targeting the gastrointestinal tract with genetic immunotherapies is becoming a promising strategy [120–122]. However, it is challenging to deliver siRNA and pDNA due to extracellular and intracellular obstacles. In a previous study, chitosan was used to modify MTG-based NPs to remain retained in the gastrointestinal tract, which allowed them to circumnavigate many barriers in the intestinal epithelium [123]. By modifying the MTG/siRNA/pDNA NPs with Gly and Man ligands, they could successfully cross enterocytes and M cells. Approximately 27% of the MTG/siRNA/pDNA NPs traversed across the ileum at a rate ex vivo [120].

The main challenge for vaccine delivery is to target the LN directly with antigens to activate the immune cells and cytokines in the LN, initiating an adaptive immune response [123, 124]. Presently, traditional oral vaccines lack the ability to prevent gastrointestinal-related infections. In contrast, oral administration of the MTG/siRNA/pDNA NPs led to a robust and systemic immune response because the MTG/siRNA/pDNA NPs that were delivered orally were passively transported into the MLN via the M cells at the site of Peyer's patch and by the pathway of enterocytes [120]. The MTG/siRNA/pDNA NPs could enter lymphatic circulation and then become allocated to other lymphoid organs to interact with macrophages and DCs. The MTG/siRNA/pDNA NPs could also transit into the thoracic duct and bind to macrophages in the blood and spleen [120]. As the MTG/siRNA/pDNA NPs entered the macrophages via endocytosis, the MTG/siRNA/pDNA NPs became oriented into the ER and Golgi apparatus and were able to escape lysosomes, leading to efficient transfection of siSIRPa and pMUC1. After the in situ transfection of the macrophages, this brought about the gene silencing of siSIRPa and pMUC1, which could be distributed throughout the tumor and the body, stimulating more systemic and local antitumor responses of the immune system [125, 126]. The immune response produced from the orally administered MTG/siRNA/pDNA NPs was more potent than the responses induced by subcutaneous injection because oral delivery caused more efficient transit through the lymphatic system with increased targeting of APCs. Injections of vaccines into the lymphatic vessels can initiate more robust immune responses than other routes of vaccination [127, 128]. However, these types of vaccination routes are challenging to achieve because of the difficult positions of orientation needed for this approach to function. Oral transport of genes could lead to a potential alternative for reaching lymphatic vessels with vaccines.

After oral delivery of a vaccine, the gene-loaded nanoparticles (NPs) used to target the M cells can successfully become transported to the gut-associated lymphoid tissue (GALT) by crossing the M cells into the Peyer's patch of the ileum [128]. Enterocytes can also be used for transport into the lymphatic system. The NPs can become engineered to specifically cross the ileal enterocytes and enter into the lacteal [129]. The NPs in the Peyer's patches and the lacteal merge into the mesenteric lymph nodes (MLNs) and then disperse to other lymphoid organs via lymphatic transport and circulation, which stimulates entry into APCs, including DCs and macrophages [130]. In a previous study, siRNA was loaded into mannose (Man)-modified chitosan-based NPs that were delivered to M cells and macrophages [131, 132]. Many studies have also demonstrated that bile acid (BA)-based NPs tend to circulate in the lymphatic system after they transit the ileal epithelium via the apical sodium-dependent bile acid transporter (ASBT) [133, 134]. Thus, it is preferable to design NPs with different properties specific for lymphatic transport for crossing the M cells and enterocytes to enhance accumulation in the LN with increasing targeting of the macrophages by orally administered pMUC1 and siSIRPa, which can optimize the antitumor immunotherapeutic effects [135, 136].

### PELNVs provide colon-targeted delivery

PELNVs can successfully perform targeted drug delivery to the colon after oral administration because PELNVs innately optimize the bioavailability of bioactive molecules by maintaining the stability and security of the loaded cargo [85, 86]. A study demonstrated that PELNVs are resistant to the lysis of detergents. PELNVs can resist degradation by digestive enzymes, which assist in avoiding damage to their cargo by the volatile gastric regions of the intestines to become securely delivered to the colon. PELNVs loaded with doxorubicin achieved efficient uptake by Colon-26 tumor cell lines. The PELNV loaded with doxorubicin effectively suppressed the growth of Colon-26 tumor cells. The ginger-derived ELNVs were able to inhibit oxidative stress and repress the release of proinflammatory cytokines, which produced a significant antitumor effect [32]. The ginger-derived nanovectors may have induced the release of doxorubicin at an acidic pH, thereby taking advantage of the acidic tumor microenvironment. This action by the ginger-derived nanovectors, promoting a pH response-to-release of doxorubicin into the tumor may have also limited the negative side effects of doxorubicin. PELNVs are more efficacious than synthetic liposomes because PELNVs have higher levels of safety, act as apoptotic agents that are pH-reliant during drug release and are more biocompatible [108, 109]. The intestinal lining of mucosa includes multiple types of epithelial cells, such as discoid cells, goblet cells, and a lamina propria that underlies this intestinal mucosa.

The goblet cells form the mucus layers that cause microbes to cease from attaching to the intestinal epithelial cells. The discoid cells ensure the the mucus layers are sterile by producing antimicrobial peptides, and the lamina propria holds the immune cells, such as the antigen-presenting cells of dendritic cells [DCs], B cells, and T cells. Because the immune system in the intestines contains the largest source of immune cells in the human body, at approximately 70%, and the intestines consists of many antigen-presenting cells in its lamina propria [137], the PELNV-based vaccine orally administered through gavage would deliver antigens into the immune system of the gastrointestinal tract. Cancer vaccines mainly administered intramuscularly and subcutaneously are being examined in most clinical trials [138]. The cancer vaccines administered subcutaneously and intramuscularly rely on the available draining lymph nodes and a limited number of antigen-presenting cells contained in the subcutaneous and muscle tissues. Oral cancer vaccines can produce strong immune responses against tumors through intestinal immunity [139]. Cancer vaccines delivered through oral administration are safer than injection, but the effectiveness of oral cancer vaccines have been continuously limited by degradation in the gastrointestinal tract environment and the barriers presented in the intestinal epithelium that further reduces their effectiveness.

However, PELNVs used to deliver cancer vaccines through oral administration can provide stability when transiting through the different pH values present in the gastrointestinal tract and can permeate across the barriers of the intestinal epithelial cells via endocytosis to present antigens to immune cells in the lamina propria. Utilizing PELNVs can effectively deliver orally administered vaccines since they inhibit viral infection, stimulate immune responses, and play a therapeutic role during the development of disease. Additionally, there are a few similar properties shared between exosomes and viruses, such as size, biochemical consistency, transferring biochemical mechanisms, organizing host cell entry, and biogenesis. Researchers have investigated the therapeutic potential of PELNVs to present antigens as a safe vaccine design [140]. PELNVs can carry cargo, such as antigens, and naturally deliver the antigens to antigen presenting cells [33, 140, 141]. A preclinical study published in the Journal of Biological chemistry [142] showed that PELNVs loaded with mRNA effectively delivered many mRNAs, which provides additional support for developing PELNVs loaded with mRNA to deliver vaccines and other therapeutics. Because PELNVs are stable, highly permeable in the vascular system, soluble, and have an increased capacity for biodistribution, PELNVs are favorable drug delivery systems for vaccines [143]. Many in vivo studies have been conducted to analyze the level of toxicity and immunogenicity of PELNVs [144], and these studies found that PELNVs can effectively and safely be applied in developing and designing vaccines. The PELNVs can carry and preserve the antigen conformation and increase the antigens' access to all organs through their transit via bodily fluids, offering a significant advantage versus other drug delivery systems, such as viral vectors and synthetic lipid nanoparticles [145, 146]. PELNVs can efficiently deliver vaccines because they are effective antigen-presenting agents with increased levels of biosafety.

### The use of PELNVs to deliver cancer vaccines via oral administration

Exosomes extracted from tumor cells can deliver therapeutics that stimulate a potent antitumor immune response [147]. Exosomes used to deliver vaccines were effective against infectious diseases. Exosomes isolated from natural immune and tumor cells can deliver cancer vaccines [148]. Exosomes have MHC molecular complexes on their surfaces that help to orient and produce antitumor immune responses from immune cells. Many preclinical studies have demonstrated the significant use of exosomes in the immunotherapeutic treatment of cancer [149, 150]. The exosomes used for delivering the anti-survivin immunotherapy increased the survival of the patients by lowering the levels of CD9 + /SVN + and CD9 + /GFAP + /SVN + [151]. Exosomes isolated from tumor cells consist of DNA that can activate immune cell responses via the STING/cGAS mechanism and pathway, which may control the internal immunity of the tumor through the process of checkpoint immunotherapy [152]. Exosomes obtained from DCs found in cancer patients are safe for use in cancer immunotherapies in a few clinical trials. DC-based exosomes are more biostable and bioavailable with more frugality in cost [153]. In phase I of a clinical trial (NCT01159288), the researchers used autologous DC-based exosomes to deliver a vaccination against a metastatic melanoma that proved to be a safe form of drug delivery. However, there were no observed immune responses from CD4 + or CD8 + T cells, and this less effective result requires further examination of the distribution of antigens by the exosomes [154].

Exosomes acquired from DCs augment the lysis conducted by NK cells and activate responses from antigen-specific T cells in cancer patients [155]. In a phase II clinical trial, IFNγ DC exosomes delivered therapeutics to cancer patients [156]. The researchers of this clinical trial reported that DC-based exosomes increased the antitumor immune responses of NK cells in patients [157]. DC-based exosomes were used to deliver NK-based therapeutics [158] that generated a synergistic immune response of eliminating NK-reliant tumors. DC exosomes altered to express an MHC-II molecule with or without MHC proteins showed the vast potential of stimulating the continuous production of DC exosomes that can lower the costs of cancer therapies and decrease the time of cell culture. DC exosomes effectively eliminated glioblastomas in the brains of mice. Exosomes have delivered immunotherapeutic cancer vaccines. Presently, clinical trials have shown much significance for providing evidence for the usefulness and safety of administering exosomes extracted from autologous sources; however, there are more issues of safety with the use of exosome-based extracts. More preclinical and clinical tests are needed to further investigate protocols for acquiring large quantities of exosomes from sources and for examining exosome biocompatibility [159, 160]. Since the morphology of exosomes is smaller in size and has a more uniform shape, exosomes can efficiently escape clearance by the mononuclear phagocyte system (MPS), which extends their time of circulation while incurring a higher level of cell-to-cell communication.

Plant-derived EVs (PEVs) are effective nanocarriers of mRNA vaccines when formulated into oral capsules for ease of use. Because EVs have many innate abilities to protect and deliver cargo to their cell target sites, EVs are becoming a promising technology with immense potential. EVs do not cause toxicity, and cells can easily uptake these EVs [161]. There is ample evidence that proves that human cell-derived EVs are an efficient means of encapsulating therapeutics [162]. The antigens carried by EVs were more sufficiently presented than free-soluble antigens to stimulate a robust cytotoxic T-cell response [163]. However, since human cell-derived EVs are expensive and difficult to produce, they are not frequently used for vaccine design and development. Plant-derived EVs or PELNVs are alternatives to human-cell-derived EVs for mRNA vaccine delivery. PELNVs are promising for delivering gene-based vaccines since their production is frugal and with a higher yield, they do not cause toxicity, they prevent damage to nucleic acid cargoes from enzymes or stress, and they are modifiable [80, 164–166]. PELNVs isolated from plants are resistant to the gastrointestinal tract, have stability, and are without immunogenicity [167]. This review attempted to describe the potential for using plant-derived EVs to deliver cancer vaccines that can initiate an immune response. For example, in a previous study, the researchers used EVs isolated from oranges to deliver mRNA-based vaccines that were formulated for oral capsules. The orange-derived EVs protected and safely delivered the mRNA vaccine for SARS-CoV-2 S1.

Orange-based EVs (oEVs) can protect nucleic acid cargo from enzymes such as RNase and digestive enzymes and prevent damage from an acidic gastric pH [167]. The study showed that orange-derived EVs can stimulate a robust humoral and cellular immune response after oral, intranasal, and intramuscular injection [167]. In the study, researchers loaded orange EVs into gastric-resilient capsules that became disintegrated in the first intestinal tract, where a larger portion of the immune system is present to block premature gastro-diffusion [168]. After oral administration of the mRNA vaccine carried in orange EVs, the oEV carriers of the mRNA vaccine triggered strong cellular and humoral immune responses in rats, which produced neutralizing antibodies of IgM, IgG, and IgA. The produced IgA is a part of the adaptive immune response after mucosal vaccination [169, 170]. Mucosal vaccination also triggers a high serum level of IgM and IgG, which provides a more systemic form of immunity and protection [171]. Vaccines delivered through the oral route can directly target the site where most of the immune system exists, which can trigger an immune response. In their study, the immunization of the rats did not prompt toxicity, which the behavioral and histological tests confirmed. Vaccination of rats with orange-derived EVs (oEVs) carrying mRNA vaccines for SARs activated Th-1 cytokine secretion, releasing IFN-γ and IL-2. The oEV-loaded S1 mRNA vaccine produced CD3 + T-cell responses in the spleen while expressing the activation marker CD25 on CD3 + and CD4 + lymphocytes. Memory T cells also proliferated and expressed activation following vaccination [172]. Mucosal vaccination against SARS-CoV-2 induced an increased response from CD4 + T cells in mice [173, 174].

**Fig. 3:**
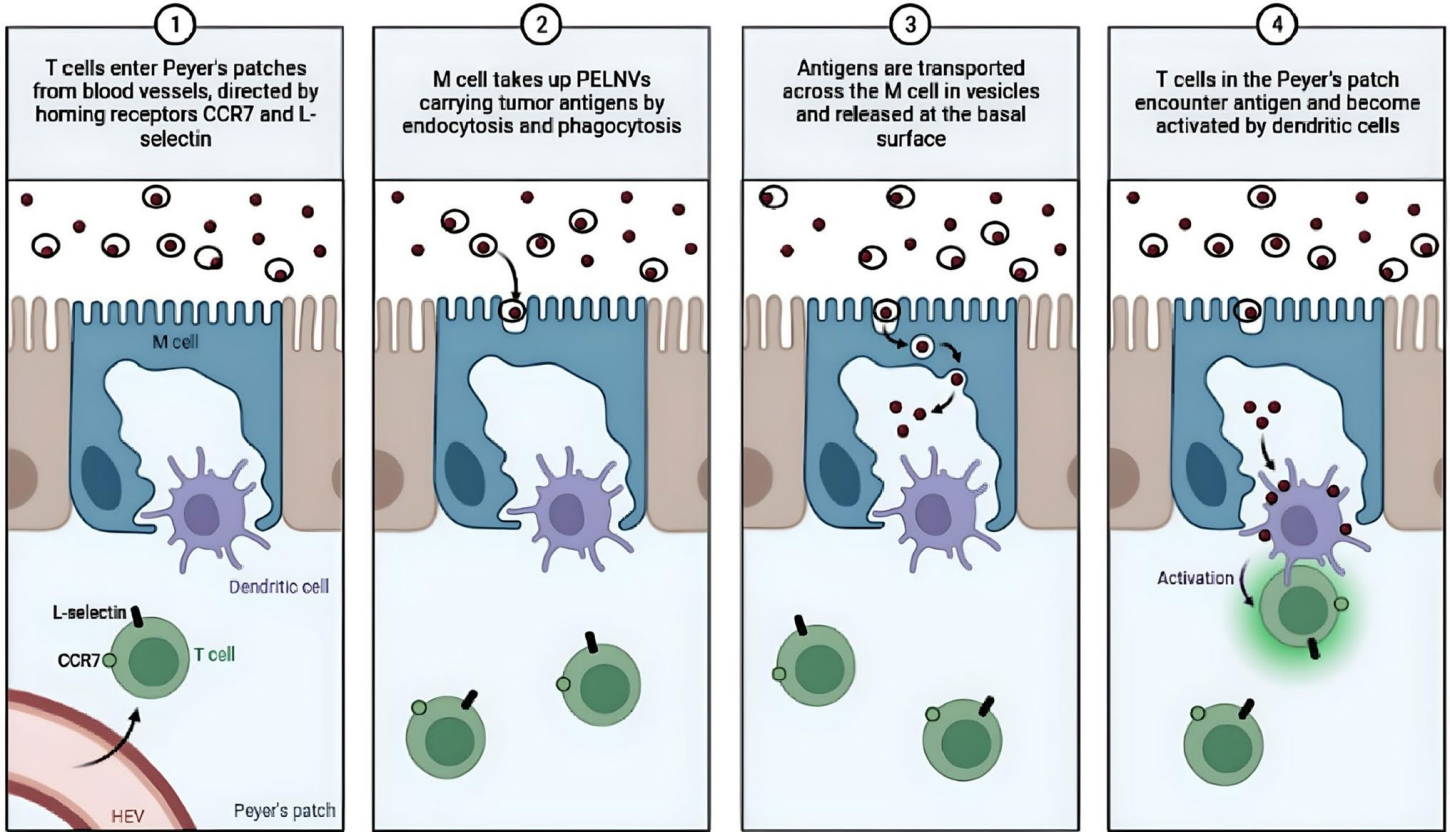
Cancer vaccines delivered by PELNVs into the intestinal immune system. 1. PELNVs deliver tumor antigens into the colon. 2. The PELNV-encapsulated tumor antigen binds to the M cells. 3. The M cell performs endocytosis of the PELNV-based tumor antigen. 4. The tumor antigens are transferred into Peyer’s patches to be presented by dendritic cells to T cells

**Fig. 4:**
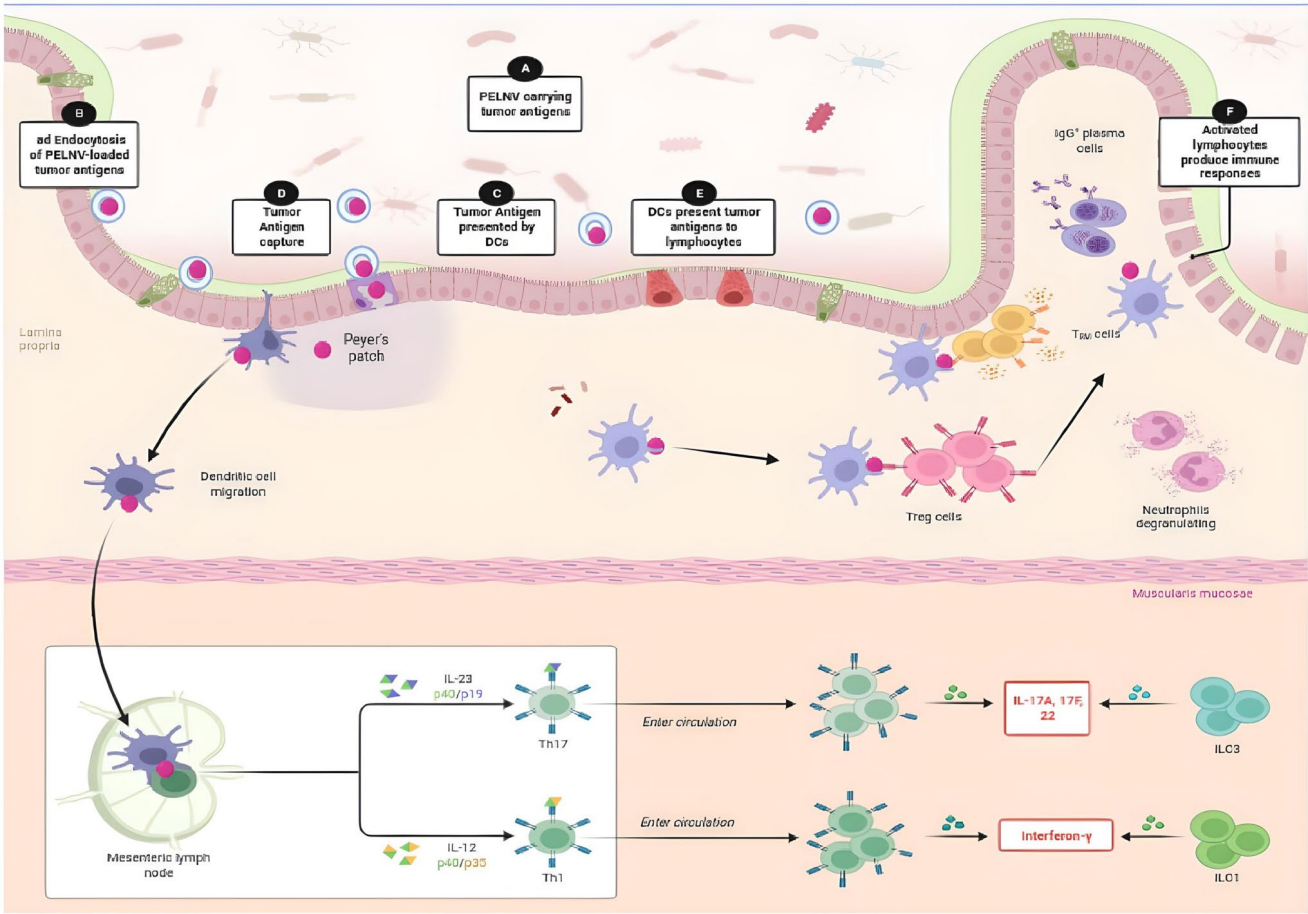
PELNV-loaded tumor antigens in Peyer’s patches and lymph nodes. **A** PELNV-loaded tumor antigens are delivered into the colon. **B** The PELNV-loaded tumor antigens undergo endocytosis with M cells. **C** The tumor antigens are presented by the dendritic cells. **D** The tumor antigens are captured. **E** Dendritic cells present tumor antigens to lymphocytes. **F** The lymphocytes become activated and produce immune responses

**Fig. 5:**
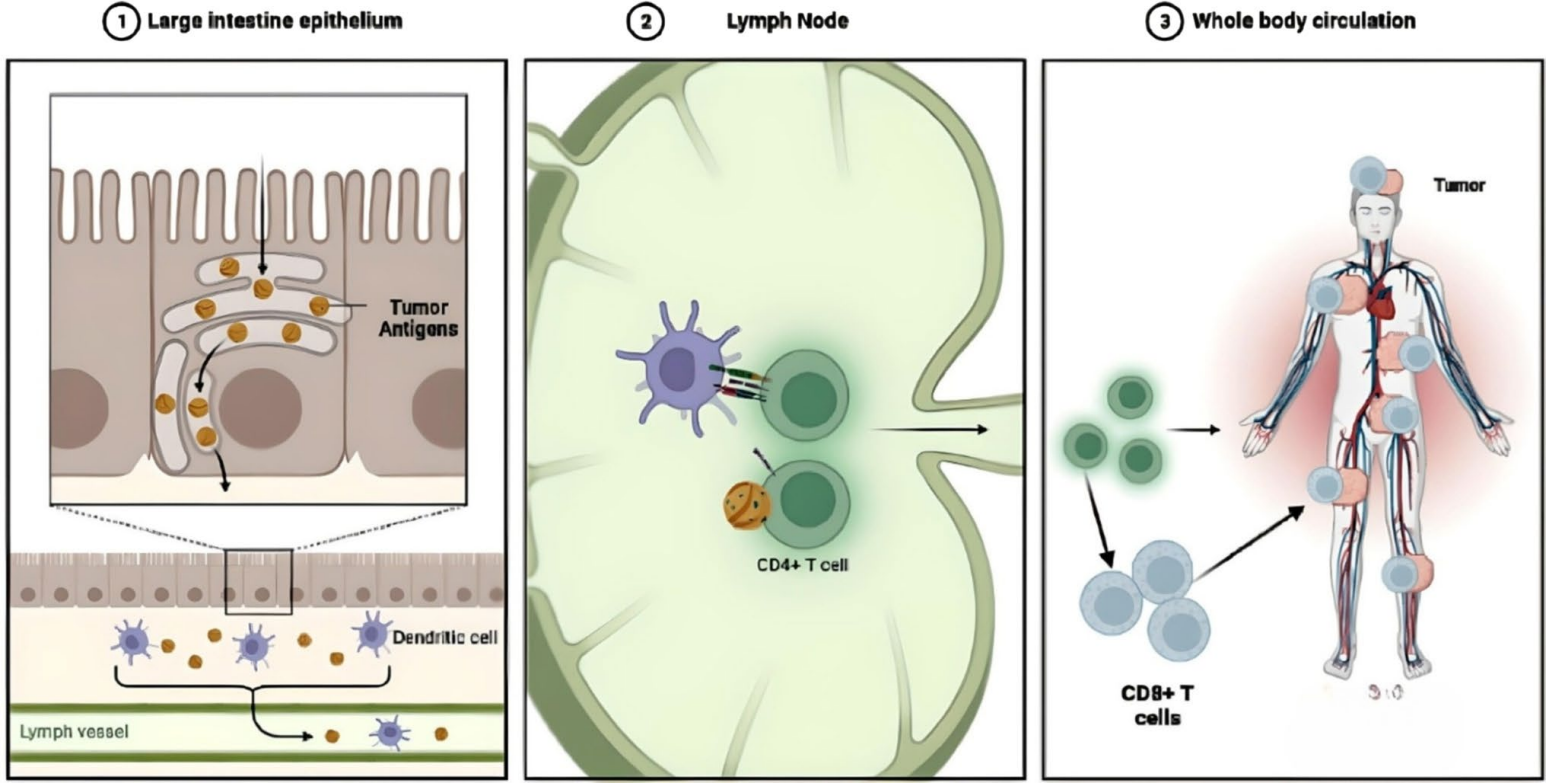
The tumor antigens delivered by PELNV produce immune responses and eliminate tumor cells. 1. The tumor antigens delivered into the large intestines are processed by M cells in the large epithelium of interest, recognized by dendritic cells, and then transported to the lymph nodes via lymph vessels. 2. Dendritic cells present tumor antigens to CD4 + T cells in the lymph nodes. 3. The CD4 + T cells activate the CD8 + T cells that recognize the specific tumor cells expressing the targeted tumor antigens, and then the antigen-specific CD8 + T cells eliminate the tumor cells

Extraction of EVs from plants may be a potentially effective drug delivery system for delivering oral vaccines for enhanced mucosal saturation [173, 174]. The SARS-CoV-2 S1 mRNA-based oEV vaccine is a model for the future design of vaccines against other infectious diseases and demonstrates the immense potential of plant-derived EVs [167]. The study showed that plant- derived EVs can be an effective nanocarrier of mRNA vaccines, which is a mechanism that has many applications in the clinical setting. Through oral administration, the largest storage of the immune system that is in the intestines can become efficiently targeted [167]. The oral delivery of a vaccine into the intestines allows for a more significant resulting immunization [167]. Oral administration does not require the use of needles for intravenous injections, which can advance to a larger-scale adherence to vaccination initiatives by allowing an easier, speedier, and more frugal cost with less pain from vaccinations intravenously injected. Likewise, an orally administered cancer vaccine delivered through the use of PELNVs would produce similar effects and immune responses as the DC exosomes and the oEV-delivered SARs-CoV-2 S1 mRNA vaccine. The tumor antigen would be transported through PELNVs, such as oEVs, into the colon, and then the PELNVs would interact with the M cells via a process of endocytosis (Fig. 3). The tumor antigen can traverse across M cells and other enterocytes into the Peyer's patches (Fig. 3 and Fig. 4). Tumor antigens present in Peyer's patches encounter dendritic cells, and the dendritic cells present antigens to immune cells such as T cells and B cells (Fig. 4). The recognition of tumor antigens by T cells and B cells activates the migration of lymphocytes to the lymph nodes (Fig. 4 and Fig. 5). B cells also produce IgA and IgG antibodies in the Peyer's patches. IgG enters the blood circulation. In the lymph nodes, the APCs present the tumor antigens to helper T cells, which become activated and produce cytokines, interleukins, and IFN-γ (Fig. 4). The APCs present the tumor antigens to the B cells (Fig. 4).

The B cells initiate and generate IgG and IgM antibodies that bind to the epitopes of the tumor. The release of interleukins and cytokines by helper T cells stimulates the increased activity of cytotoxic lymphocytes, such as CD8 + T cells, to begin attacking and eliminating the tumor (Fig. 5). Edible PELNVs used as nanovectors, which were coated with inflammatory-like receptors extracted from leukocytes, successfully treated the inflamed tissues of inflammatory-induced mouse models and was patented with a U.S Patent Application Publication listed as No. 2017/0035700. A third generation edible PELNV-nanovector coated with segments from plasma membranes and used to eliminate tumors with cancer cells were patented by Huang-Ge Zhang in the U.S Patent and Trademark Office with a publication number of US 2021/0236612 on Aug. 5, 2021 [175]. These third generation edible PELNV-nanovectors stimulate immune responses to attack and eliminate tumors by presenting tumor and cancer-related antigens to antigen presenting cells (APCs), and then producing immune responses to act against the tumor and cancer-related antigens. The patent of US 2021/0236612, for the third generation edible PELNV-nanvectors used to deliver tumor-and cancer-associated antigens to kill tumors and/or cancer cells, is based on the research study results of mice injected with melanoma cells and then given edible PELNV-nanovectors carrying miR18a, which was the tumor-associated antigen, to gavage. miR18a is an onco-microRNA that is increasingly expressed in many different tumors and in progressively advanced tumors. The study found that the lungs of the mice after the treatment with the edible PELNVs loaded with miR18a showed significant reductions in lung metastases when compared against the control groups.

### Challenges of utilizing PELNVs for therapeutic drug delivery

PELNVs can act as mediators of cell-to-cell communication through the transference of different proteins, lipids, and genetic information to modify the phenotypic characteristics and actions of the targeted cells. PELNVs are now being considered and utilized in many biological and pathologic mechanisms among the cells of species other than from the cells of plants [33]. However, it is still not known whether these forms of communication with PELNVs are specific. Even so, the application of PELNVs as effective drug delivery systems for nanotherapeutics to deliver proteins, drugs, and nucleic acids to traverse many different biological obstacles is gaining much attention from the scientific community [33]. However, the cellular and molecular processes that control the biogenesis, release, and actions of PELNVs are still unknown. Furthermore, the processes of isolation PELNVs expend much time, and these processes require much revision to translate PELNV use into commercial and clinical settings. The analyses used to examine the quality of the PELNVs require much more normalization and standardization. Currently, there are no regulations present about how to classify or approve PELNVs [33]. There is also no agreement about the general propensities of PELNVs to become internalized for delivery to recipient cells and how the cells uptake the PELNVs [33]. Another limitation is that PELNVs have a limited capacity for carrying drug cargo that has a high mass [33].

A technique called membrane fusion could create a hybrid between synthetic liposomes and PELNVs to increase the capacity for PELNVs to carry a high quantity of cargo. The synthetic liposomal portion of the hybrid can provide additional modifiable physicochemical properties. This hybrid could resolve many of the immunogenic and cytotoxic issues of the synthetic liposome with a combination with small-sized PELNVs with their ability to cross many challenging biological barriers and to escape detection by the mononuclear phagocyte system (MPS) [33]. We also have a limited understanding and knowledge of the diversity, physiology, and cargo-delving capabilities of PELNVs [33]. It is still essential to understand whether cellular uptake of PELNVs occurs through macropinocytosis or micropinocytosis via receptor-mediated pathways. Researchers may need to determine how each of these processes leads to the delivery of cargo into recipient cells. Understanding the mechanisms of macropinocytosis and micropinocytosis for cargo delivery by PELNVs will help in regulating the uptake of PELNVs with the subsequent delivery of their cargoes, which can have significant implementations in the clinical setting [33]. PELNVs have immense potential for application as an effective drug delivery system because of their innate biochemical properties, and they are highly bioactive. However, loading drugs into these nanovesicles may cause a negative interaction between their bioactive segments and the loaded drug molecules. Hence, more investigations are needed when designing methods for loading exogenous drug compounds into these nanovesicles to observe any negative interactions. Bioactive compounds eliminated from the PELNVs before loading the drug molecules should not damage the size, morphology, or bioactivities of the PELNVs.

The therapeutic effects of PELNVs mostly occur through their innate capability for biodistribution. The surfaces of PELNVs can be modified to ensure targeted delivery to difficult-to-reach sites of tumors. Second, PELNVs applied as a drug delivery system in the future can transit across the BBB because they can innately transport across the BBB while resolving toxicity issues and avoiding clearance by the MPS, in which many other nanocarriers cannot avoid clearance by the MPS without causing much toxicity. Third, PELNVs could become hybrids designed to form combinations with magnetic nanoparticles that have photothermal potential. These types of nanohybrids for drug delivery could widen the discovery of more theranostic functions of targeted drug delivery amalgamated with hyperthermia under the control of a magnetic field [33]. A preclinical study published in the Journal of Biological Chemistry [142] determined that mRNA-loaded PELNVs can effectively deliver mRNAs and can be used in vaccine designs. Other preclinical studies include: PELNVs used to orally administer miRNAs to reduce miRNA target gene expression [176], aloe green vesicles loaded with indocyanine to reduce the growth of melanomas [177], cabbage PELNVs loaded with miR-184 and doxorubicin to decrease cell tumor growth [178], ginger nanovesicles loaded with doxorubicin to suppress tumor growth in vivo [179], and PELNVs loaded with siRNA of CD98 and orally administered to inhibit CD98 expression in the tissues of the colon [180], ginger vesicles engineered with folic acid were loaded with siRNA and administered intravenously to suppress tumor growths in a model of murines [181].

Ginger nanovesicles were loaded with 6-shogaol and orally administered for its strong regenerative effects [182]. Grapefruit nanovesicles encapsulating anti-Stat3 inhibitors (JSI-124) were administered intranasally to mice to inhibit expression of Stat 3 [79]. Nanovesicles extracted from grapefruits were also used to carry doxorubicin to target glioma tumors in vivo [183], and the grapefruit nanovesicles were studied for their capacity to uptake and eliminate the proteins of HSP70 and BSA [184]. Nanovectors engineered from grapefruit-PELNVs were coated with inflammatory-associated receptor-enhanced membranes from leukocytes for their increased ability to bind to tumor tissues [185]. Grapefruit vesicles have also been loaded with miR-17 to target brain tumors [186] and extracted to deliver miR18 for inhibiting liver metastasis [187]. Nanovesicles extracted from lemons have been loaded with doxorubicin to treat cells resistant to doxorubicin and to eliminate tumors [188]. There are plant-based vaccines, utilizing PELNVs, against influenza that are currently undergoing clinical trials and show great potential of effectiveness with increased safety [77]. There is an active clinical trial listed as NCT01294072 that has loaded PELNVs with curcumin to treat colon cancer, but the clinical trial has not recruited study participants [189]. A second active clinical trial listed under NCT01668849 is being conducted with PELNVs derived from grapes for treating chemotherapy and radiation-related oral cancer [190]. A third active clinical trial labeled NCT03493984 has extracted PELNVs from ginger to examine the potential of the PELNVs to treat insulin resistance and the inflammation caused by polycystic ovary syndrome [191].

## Conclusion

PELNVs can provide improved drug delivery systems for cancer immunotherapies. This review serves to offer evidence for the immense potential of PELNVs to deliver cancer vaccines via oral administration. Because PELNVs are resilient to changes in pH and can prevent the degradation of their cargoes by the gastric enzymes present in the gastrointestinal tract, PELNVs are auspicious in delivering oral therapeutics for treating cancer. Specifically, PELNVs could provide a more targeted delivery of cancer vaccines via an oral route of administration since they can escape clearance by the MPS, have less immunogenicity, and can perform effective endosomal escape due to their innate bioactive capabilities. When PELNV-based cancer vaccines delivered through oral administration can efficiently reach the colon and become endocytosed by colonic M cells, the tumor antigens are released into Peyer's patches linked with DCs, and then the DCs present the tumor antigens to the immune cells that traverse to the LNs to release more immune responses for the elimination of the tumor by antigen-specific cytotoxic T cells. However, many challenges persist for the complete application of PELNVs as drug delivery systems in commercial and clinical settings.

The challenges include the lesser capacity of the PELNVs for carrying high quantity cargos, negative interactions between innate present biomolecules of the PELNVs with the therapeutic cargos, and the extended time of extraction required to produce the purest forms of PELNVs. The use of forming hybrids between synthetic liposomes and PELNVs could increase their capacity for higher quantity cargos. The biomolecules innately present in unloaded PELNVs can address the issue of negative interactions between the biomolecules and the therapeutic cargo. Additionally, protocols need more generalization of the processes for PELNV isolation to ensure a reduction in the extended time needed for extracting PELNVs. Since the bioavailability and biocompatibility of PELNVs are still unknown, future research should investigate and discover improved methods for regulating the general production and use of PELNVs for drug delivery. Hopefully, this review may add to the growing body of literature on PELNVs, in which this review describes PELNVs as efficient drug delivery systems for cancer vaccines when administered via an oral route.

## Data Availability

Not applicable.
